# Solid-State Characterization of Different Crystalline Forms of Sitagliptin

**DOI:** 10.3390/ma12152351

**Published:** 2019-07-24

**Authors:** Nayana C. F. Stofella, Andressa Veiga, Laiane J. Oliveira, Elisa F. Montin, Itamar F. Andreazza, Marco A. S. Carvalho Filho, Larissa S. Bernardi, Paulo R. Oliveira, Fábio S. Murakami

**Affiliations:** 1 Departamento de Farmácia, Universidade Federal do Paraná, Av. Pref. Lothário Meissner, 632-Jardim Botânico, Curitiba 80210-170, Paraná, Brazil; 2 Escola de Ciências da Saúde, Universidade Positivo—UP, R. Prof. Pedro Viriato Parigot de Souza, 5300, Curitiba 81280-330, Paraná, Brazil; 3 Programa de Pós-Graduação em Ciências Farmacêuticas, Universidade Estadual do Centro-Oeste (UNICENTRO), Rua Simeão Camargo Varela de Sá, 03-Vila Carli, Guarapuava 85040-080, Paraná, Brazil

**Keywords:** crystallinity, characterization in solid state, physicochemical properties, solubility, bioavailability

## Abstract

Sitagliptin is an inhibitor of the enzyme dipeptidyl peptidase-4, used for the treatment of type 2 diabetes mellitus. The crystal structure of active pharmaceutical solids determines their physical and chemical properties. The polymorphism, solvates and hydrates can influence the free energy, thermodynamic parameters, solubility, solid-state stability, processability and dissolution rate, besides directly affecting the bioavailability. Thus, the physicochemical characterization of an active pharmaceutical ingredient is required to guarantee the rational development of new dosage forms. In this context, we describe herein the solid-state characterization of three crystalline forms of sitagliptin: sitagliptin phosphate monohydrate, sitagliptin phosphate anhydrous and sitagliptin base form. The investigation was carried out using differential scanning calorimetry (DSC), thermogravimetry (TG)/derivative thermogravimetry (DTG), spectroscopic techniques, X-ray powder diffraction (XRPD) and morphological analysis by scanning electron microscopy. The thermal analysis revealed that during the dehydration of sitagliptin phosphate monohydrate (Tpeak = 134.43 °C, ΔH = −1.15 J g^−1^) there is a characteristic crystalline transition event, which alters the physicochemical parameters of the drug, such as the melting point and solubility. The crystalline behavior of sitagliptin base form differs from that of sitagliptin phosphate monohydrate and sitagliptin phosphate anhydrous, mainly with regard to the lower temperature of the fusion event. The melting point (Tpeak) values obtained were 120.29 °C for sitagliptin base form, 206.37 °C for sitagliptin phosphate monohydrate and 214.92 °C for sitagliptin phosphate anhydrous. In relation to the thermal stability, sitagliptin phosphate monohydrate and sitagliptin phosphate anhydrous showed a slight difference; however, both are more thermostable than the base molecule. Therefore, through this study it was possible to establish the most suitable crystalline form of sitagliptin for the development of a safe, effective and appropriate pharmaceutical dosage form.

## 1. Introduction

**Figure 1:**
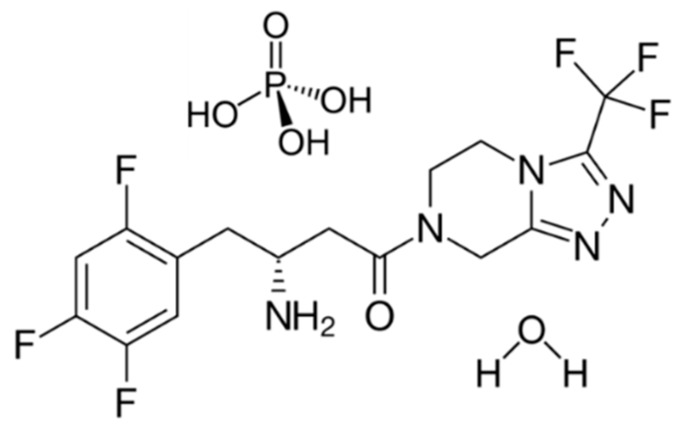
Chemical structure of sitagliptin phosphate monohydrate.

Sitagliptin (Figure 1) is an inhibitor of the enzyme dipeptidyl peptidase-4 (DPP-4) and it consequently inhibits the degradation of incretin hormones, like glucagon-like peptide-1 (GLP-1) and glucose-dependent insulinotropic peptide (GIP), resulting in increased insulin secretion and inhibited glucagon release by the beta and alpha cells of the pancreas, improving glycemic control [1,2].

Sitagliptin (7-[(3R)-3-amino-1-oxo-4-(2,4,5-trifluorophenyl)butyl]-5,6,7,8-tetrahydro-3- (trifluoromethyl)-1,2,4-triazolo[4,3-a]pyrazine) is an effective anti-glycemic drug used for the treatment of type 2 diabetes mellitus (T2DM) [3]. The original product is marketed by Merck & Co., under the trade name Januvia, as sitagliptin phosphate monohydrate, approved in 2006 by the Food and Drug Administration (FDA) [4,5].

Sitagliptin phosphate monohydrate (STG) is white to off-white, crystalline, non-hygroscopic, soluble in water and *N*,*N*-dimethylformamide, slightly soluble in methanol and very slightly soluble in ethanol, acetone and acetonitrile. It has the molecular formula C_16_H_15_F_6_N_5_O∙H_3_PO_4_∙H_2_O and the molecular weight is 523.32 g mol^−1^ [6]. 

The hydrate formation is of great importance in the pharmaceutical industry, considering the ubiquity of water vapor, because hydrates are frequently more stable [5]. Thus, the presence of solvates and hydrates influences the physicochemical properties of the crystals. 

A single molecule, such as sitagliptin, may give rise to a variety of crystalline forms with distinct crystal structures and physical properties. The structure of a crystal does not only determine the physicochemical properties of the active pharmaceutical ingredient (API) but also the solubility, stability, processability and bioavailability of the drug [7]. 

Solubility is one of the most important parameters in relation to achieving the desired concentration of the drug in systemic circulation in order to obtain the required pharmacological response. Poorly water-soluble drugs with a slow absorption, for instance, may show inadequate bioavailability [8,9].

The characterization of solid-state properties is a prerequisite in the development of new pharmaceutical solid dosage forms [5]. Thus, the characterization and differentiation of different crystalline forms of sitagliptin is required so that the physical properties of complex solvates and hydrates can be determined. Thermal analysis, for example, is a well-known technique for the characterization of APIs in terms of stability and structural investigations [10,11].

Sitagliptin phosphate monohydrate is relatively characterized and commonly used in the pharmaceutical industry; however, little is known about other crystalline forms of sitagliptin, such as the anhydrous and base form, including the effect of the dehydration process and the production of base form on the physical and chemical stability of this pharmaceutical hydrate. 

Few patents such as US patent application 2015/0087834 A1 [12] reported a method for preparation of sitagliptin phosphate and sitagliptin phosphate anhydrous, providing a brief and superficial description of X-ray powder diffraction (XRPD) and differential scanning calorimetry (DSC). The US 2009/0221595 A1 [13] reported the processes of preparing polymorphs forms, describing briefly X-ray powder diffraction and differential scanning calorimetry only the base form of sitagliptin. In fact, there is no studies in the literature that reports the detailed characterization these three crystalline forms.

In this context, the novelty of this work aims to provide a detailed solid state characterization of STG, sitagliptin phosphate anhydrous (STGA) and sitagliptin base form (STGB). The physical-chemical characteristics were investigated using DSC, thermogravimetry (TG), non-isothermal kinetics analysis, spectroscopic techniques (Fourier transform infrared (FTIR) and Raman), XRPD and scanning electron microscopy (SEM). 

## 2. Materials and Methods 

### 2.1. Sitagliptin Phosphate Monohydrate (STG)

The active pharmaceutical ingredient, STG with 99.9% declared content, batch 20170904, was purchased from Baoji Guokang Bio-Technology (Baoji, China). 

### 2.2. Sitagliptin Phosphate Anhydrous (STGA)

The sitagliptin anhydrous form was obtained by dehydration of sitagliptin phosphate monohydrate (STG). The process involved placing 2.5 g of STG in a vacuum oven (model 6030A VACUOTERM, São Paulo, Brazil) for 60 min at 300–400 mmHg and 150 °C.

### 2.3. Sitagliptin Base Form (STGB)

The base form of sitagliptin was obtained by dissolving 0.008 mol g^−1^ of sitagliptin phosphate monohydrate (STG) in 92.10 mL of purified water and then adding ammonia (10%, v/v) at pH 10.0. The solution obtained was poured into a separatory funnel and washed twice with 83.73 mL of ethyl acetate. The organic phase was dried with anhydrous sodium sulfate and filtered through quantitative filter paper. The filtrate was evaporated in a rotary evaporator and then dried at room temperature in a vacuum desiccator. A white solid crystal powder was obtained with a yield of 92% [12].

### 2.4. Differential Scanning Calorimetry (DSC)

The DSC analysis was performed in a Shimadzu^®^ DSC-60 calorimeter (Kyoto, Japan). Samples were analyzed in an aluminum crucible containing around 2.0 mg of sample under dynamic synthetic air atmosphere (50 mL min^−1^) with a heating rate of 10 °C min^−1^ and temperature range of 30 °C to 400 °C. The equipment was calibrated with indium and zinc (reference standards). 

The purity was determined using aluminum crucibles with approximately 2.0 mg of sample at a heating rate of 2 °C min^−1^ from 30 °C to 400 °C. The purity of the sample was measured in triplicate using TASYS software (version 1.14, Shimadzu^®^), based on the Van’t Hoff equation:

(1) $$X_{2}=\frac{(T_{o}-T_{m})\Delta H_{f}}{RT_{o}{}^{2}}$$

where the purity is determined from the molar percentage of impurities present in the sample, *X*_2_ represents the mole fraction of impurities, *T_m_* is the sample melting temperature, *T_o_* is the melting point of the pure substance (°K), R is a gas constant and Δ*H_f_* is the heat of fusion of the main component (J mol^−1^) [14,15].

### 2.5. Thermogravimetry (TG)

The thermogravimetric analysis was carried out on a Shimadzu^®^ DTG-60 thermal analyzer under a dynamic synthetic air atmosphere of 50 mL min^−1^. Approximately 4.0 mg of sample was placed in a platinum crucible and heated from 30 °C to 400 °C at a heating rate of 10 °C min^−1^.

For the non-isothermal kinetics studies, the curves were obtained using five different heating rates: 5, 10, 15, 20, 25 °C min^−1^. The kinetics parameters were determined by the Ozawa method using TASYS software. The equipment was calibrated with calcium oxalate (reference standard).

### 2.6. Thermogravimetry–Mass Spectrometry (TG-MS)

Thermogravimetry coupled to mass spectrometry was used to study the thermal decomposition of sitagliptin phosphate monohydrate. The analysis was carried out on a TA Instruments® MS Q600 SDT analyzer (New Castle, DE, USA). Approximately 2.0 mg of each sample was heated to 900 °C under a dynamic nitrogen atmosphere (50 mL min^−1^) applying a heating rate of 10 °C min^−1^. The equipment generates separate TG curves and MS spectra, analyzing the mass/charge (m/z) with respect to temperature.

### 2.7. Melting Point

The melting behavior was determined using a Mettler-Toledo MP70 melting point system (Greifensee, Switzerland). A capillary was used with a closed bottom, applying a heating rate of 10 °C min^−1^ up to a temperature limit of 400 °C.

### 2.8. Fourier Transform Infrared (FTIR) Spectroscopy

The infrared spectra were recorded on a Bruker Alpha-P FTIR spectrometer (Ettlingen, Germany) using attenuated total reflection (ATR) in the wavelength range of 3500 to 500 cm^−1^, with a nominal resolution of 4 cm^−1^ and accumulation of 32 scans.

### 2.9. Raman Spectroscopy

The Raman spectra were obtained with a WITEC-Alpha 300R confocal Raman microscope (Ulm, Germany), using a diode 3 mW source at a diffraction grating of 600 g mm^−1^, wavelength laser of 532 nm, integration time of 3 s, resolution of 5.0 cm^−1^ and accumulation of 30 scans.

### 2.10. X-ray Powder Diffraction (XRPD)

The X-ray powder diffraction patterns were obtained on a Shimadzu^®^ XRD-7000 X-ray diffractometer using a sample door of stainless steel of 20 mm with monochromatic radiation CuKα (λ = 1.5406 Å), a voltage of 40.0 kV, current of 20.0 mA, 2θ scanning angle and scan range of 2.0–40.0.

### 2.11. Scanning Electron Microscopy (SEM)

The morphological evaluation was performed by scanning electron microscopy (SEM) using a JEOL JSM-6360 LV microscope (São Paulo, Brazil). The sample was pre-metallized with gold and analyzed at low vacuum with an acceleration voltage of 15 kV at magnifications of 200×, 600×, 2000× and 5000×.

## 3. Results and Discussion 

### 3.1. Thermal Characterization 

**Figure 2:**
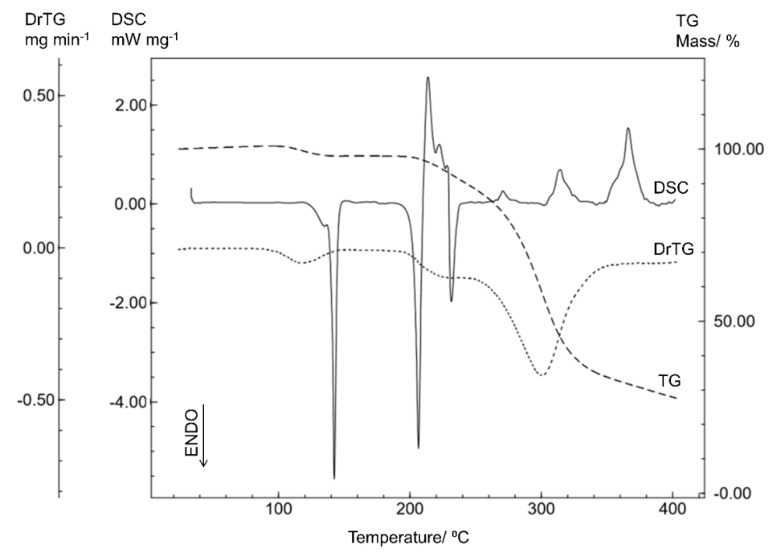
The differential scanning calorimetry (DSC) and thermogravimetry/derivative thermogravimetry (TG/DTG) curves of sitagliptin phosphate monohydrate (STG) obtained with a synthetic air atmosphere (50 mL min^−1^) and a heating rate of 10 °C min^−1^.

The thermal behavior of STG can be observed based on the DSC and TG/DTG curves in Figure 2. The DSC curve shows four endothermic events and six exothermic events. The first endothermic event corresponds to dehydration of the STG (T_peak_ = 134.43 °C; T_onset_ = 126.76 °C; ΔH = −1.15 J g^−1^), followed by a well-defined endothermic event (T_peak_ = 142.30 °C; T_onset_ = 140.61 °C; ΔH = −42.00 J g^−1^), corresponding to the crystalline transition of the sitagliptin phosphate, since no mass loss was indicated by the TG/DTG curve. The third endothermic event (T_peak_ = 206.37 °C; T_onset_ = 203.02 °C; ΔH = −104.97 J g^−1^) is characteristic of the melting process, and is in agreement with the melting point determined by capillary analysis (205.3 °C) and results reported in the literature [16]. Subsequent thermal events correspond to the decomposition process.

The first mass loss on the TG/DTG curve at between 101 °C and 136 °C (Δm = 2.9%; DTG_peak_ = 117.20 °C) confirms the dehydration event observed on the DSC curve. The second mass loss event indicates that the thermal decomposition occurs in two stages immediately after the fusion event, in the ranges of 192 °C to 243 °C (Δm = 7.9%; DTG_peak_ = 232.81 °C) and 243 °C to 385 °C (Δm = 60.4%; DTG_peak_ = 300.57 °C). Thus, the TG/DTG curve confirms that there is thermal stability up to 192 °C STG.

**Figure 3:**
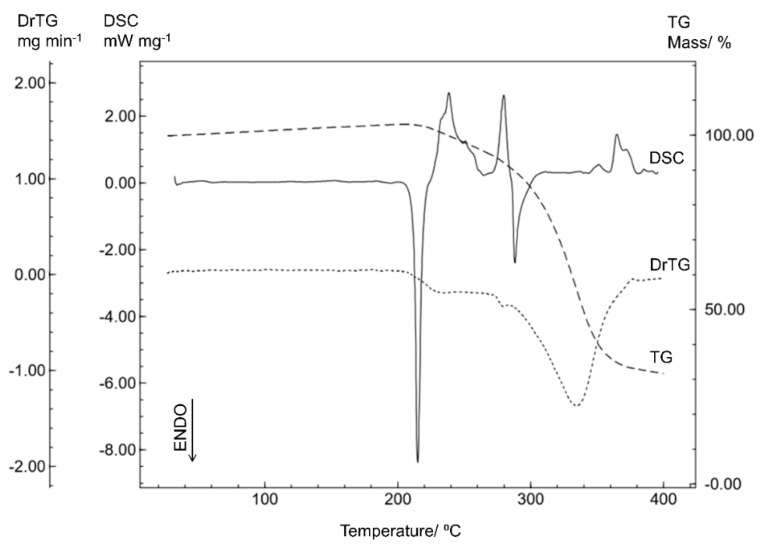
The DSC and TG/DTG curves for sitagliptin phosphate anhydrous (STGA) obtained with a synthetic air atmosphere (50 mL min^−1^) and a heating rate of 10 °C min^−1^.

In order to compare the thermal behavior of the different crystalline forms of sitagliptin, the STGA was analyzed by DSC and TG and the results are shown in Figure 3. 

The DSC curve for STGA shows two endothermic events and three exothermic events. The first is related to the melting point (T_peak_ = 214.92 °C; T_onset_ = 212.58 °C; ΔH = −104.84 J g^−1^), which was confirmed by the capillary method (212.2 °C). The subsequent events correspond to thermal decomposition, confirmed by the TG/DTG curve, indicating that thermal decomposition occurs in three steps starting at 216 °C (DTG_peak_ = 234.24 °C; DTG_peak_ = 278.74 °C; DTG_peak_ = 333.94 °C). 

It can also be noted in Figure 3 that the first endothermic event between 125 and 145 °C seen on the STG curve is not present on the STGA curve and no mass loss is indicated by the TG/DTG analysis, confirming that the water of crystallization was no longer present in this molecule. 

Although the thermal profile of sitagliptin phosphate anhydrous (STGA) is similar to that of sitagliptin phosphate monohydrate (STG), there are some differences associated with the drug crystallinity, the STGA presenting a higher melting point and a decomposition profile with three stages. 

**Figure 4:**
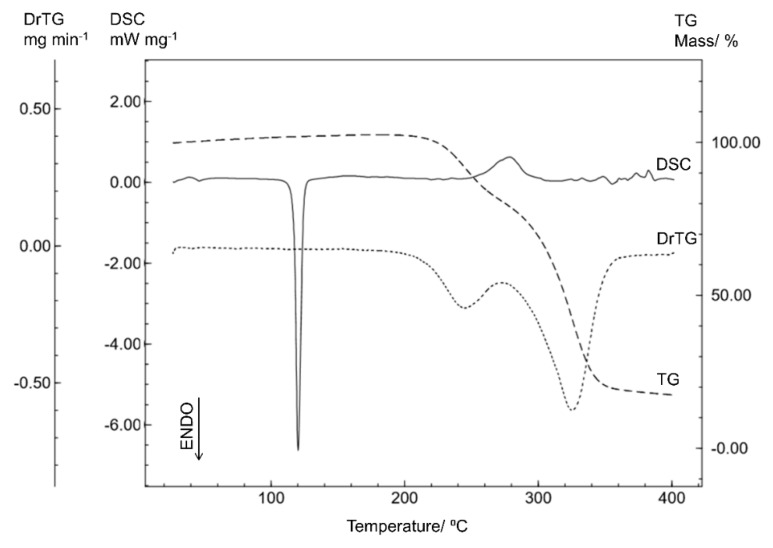
The DSC and TG/DTG curves of sitagliptin base form obtained with a synthetic air atmosphere (50 mL min^−1^) and a heating rate of 10 °C min^−1^.

The DSC curve of the sitagliptin base form (STGB) in Figure 4 shows a single endothermic event, corresponding to the melting of the compound (T_peak_ = 120.29 °C; T_onset_ = 117.81 °C; ΔH = −75.18 J g^−1^), as confirmed by the capillary analysis (121.0 °C). The TG/DTG curve indicates that the thermal decomposition takes place in two steps, in the ranges of 193 to 272 °C (Δm = 21.6%; DTG_peak_ = 244.53 °C), and 272 to 367 °C (Δm = 61.8%; DTG_peak_ = 325.33 °C), showing thermal stability up to 193 °C.

On comparing STGB with the other forms of sitagliptin, it was noted that the thermal profiles differ, especially in relation to the fusion event. In fact, for the STGB form the melting point (120.29 °C) was significantly lower than the values for STGA (214.92 °C) and STG (206.37 °C). This result suggests different crystalline forms with changes in the crystal structure. A similar behavior was reported by Murakami et al. (2009) [10] who studied the different crystalline forms of omeprazole sodium.

The analysis of the gases produced during thermal degradation can provide valuable information about the pathway of decomposition. Therefore, the thermogravimetry coupled with mass spectrometry (TG–MS) was used to analyze the gaseous products during the thermal degradation of STG.

**Figure 5:**
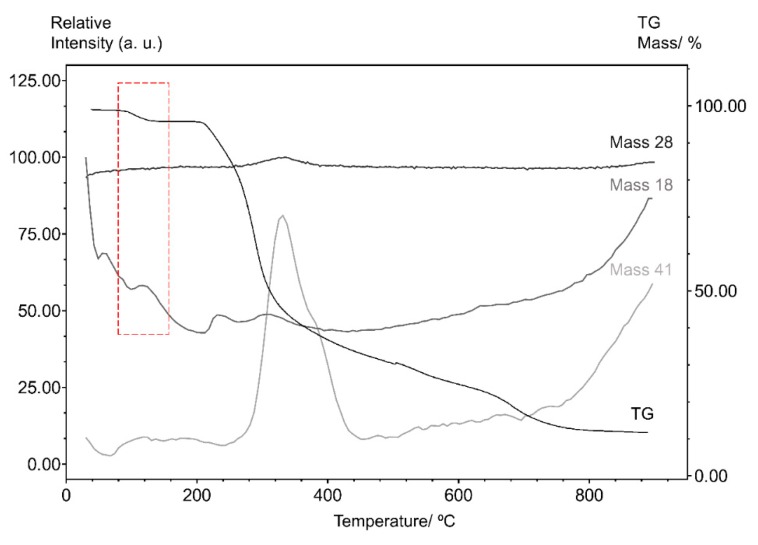
The thermogravimetric coupled with mass spectrometry (TG–MS) curves for sitagliptin phosphate monohydrate obtained with synthetic air atmosphere (50 mL min^−1^), a heating rate of 10 °C min^−1^ and 350 scans.

The TG–MS curve for STG, shown in Figure 5, provides information on the major volatile compounds released from sitagliptin phosphate monohydrate under controlled heating. The molecules most likely associated with the mass spectrum results are: H_2_O (m/z = 18), CO (m/z = 28) and C_2_H_3_N (m/z = 41).

The thermal decomposition of organic molecules, theoretically, is due to the molecular kinetic energy increasing during heating process. These include atomic oscillations that rupture the weaker chemical bonds and the cleavage occurs easier with lower orders of chemical bonds. Thermodynamically, the decomposition process also depends on the stability of the decomposition products or intermediates generated [17]. In order to understand the thermal decomposition of STG, a theoretical discussion is made from the perspective of the molecular structure.

**Figure 6:**
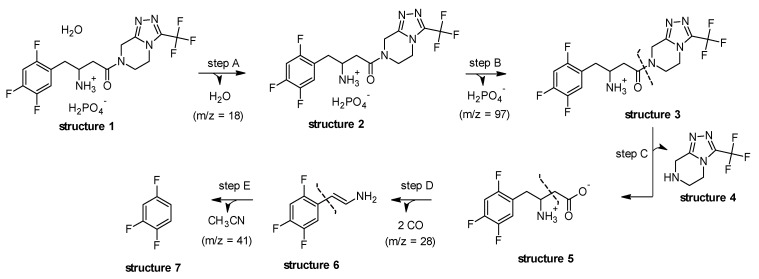
Proposal for the thermal decomposition of sitagliptin phosphate monohydrate.

According to the molecular bond order distributions of STG, the position of the chemical bonds ruptured could be deduced, and the thermal decomposition mechanism in the thermal decomposition process could be proposed and is given in Figure 6. 

As seen from the results obtained in the thermogravimetric analysis, the initial mass loss for structure 1 is associated with dehydration of the compound (step A), which was confirmed by TG–MS, since m/z = 18 (H_2_O) has a higher intensity at 117 °C. Subsequently, in structure 2 can be seen the release of phosphate group (Step B).

It can be seen from structure 3, in Figure 6, that the weakest bond of STG, no matter what kind of form, is the O=C–N bond, thus resulting in an intermediate structure 4 and 5 respectively. Therefore, the crucial step of thermal decomposition is the cleavage of the amide group as seen in step C. A similar fragmentation pattern was described by Vishnuvardhan et al. [18]. 

Furthermore, at approximately 330 °C, it is possible to observe in step D, the probable cleavage of the C–C bond in structure 5, and the release of molecules such as CO (m/z = 28). Finally, in step E, the structure 6 has been associated with release of acetonitrile (CH_3_CN; m/z = 41), since it corresponds to the most stable form of stabilization when it assumes the cleavage between the C–ring and the branching of structure 6, resulting in a final structure 7. The thermal decomposition of the compound, occurring rapidly, successively and almost simultaneously, and this process appears as a three events on the TG/DTG curve.

### 3.2. Purity Study

**Figure 7:**
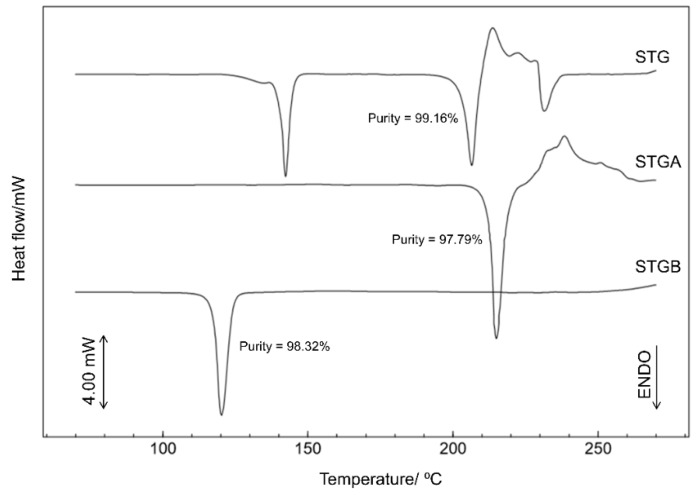
DSC curves for STG, STGA and sitagliptin base form (STGB) obtained with synthetic air atmosphere (50 mL min^−1^) and a heating rate of 2 °C min^−1^^.^

The determination of purity is based on the assumption that an impurity modifies the characteristics of a pure material, changing the endothermic melting enthalpy. The melting transitions of a pure material (100% crystalline) must be sharp, and the presence of small amounts of impurities will generate a reduction in the melting point and widening of the overall melting range [19]. Figure 7 shows the results for all samples and the calculated purity values were 99.16% ± 0.33 for sitagliptin phosphate monohydrate, 97.79 ± 0.87% for sitagliptin phosphate anhydrous and 98.32% ± 0.62 for sitagliptin base form.

Therefore, in this analysis, the melting events were observed as symmetrical and well-defined endotherms, indicating that the dehydration process and the production of the base form did not modify the purity characteristics.

### 3.3. Kinetics Analysis

In order to evaluate the stability of the STG, STGA and STGB forms, the samples were subjected to non-isothermal kinetics analysis based on five TG curves obtained at different heating rates. The effect of temperature was evaluated in terms of the thermal decomposition rate and reaction order.

Non-isothermal thermogravimetric analysis with a linear heating rate is a technique for studying the process kinetics that allows the kinetics parameters of isoconversional solid-state reactions to be determined through calculations. The Flynn–Wall–Ozawa (FWO) method is one approach to studying these kinetics parameters, where the activation energy (E_a_) is calculated by dynamic heat thermogravimetry [20,21].

The isothermal method is based on the Arrhenius equation and is given by the following equation:

(2) $$\log\;A=\log\left[{\mathrm{ZE}}_{a}/\mathrm{Rf}\left(\alpha \right)\right]-2.315-0.457\left(E_{a}/\mathrm{RT}\right)$$

where A is the heating rate, Z is the pre-exponential factor, E_a_ is the activation energy (J mol^−1^), R is the gas constant; f(α) is the integral conversion function; and T is the temperature (K). 

The natural logarithm of the heating rate (log A) against the inverse of the temperature (1/T) on the TG curves at different heating rates will give a straight line with a slope of −0.457 (E_a_/R), allowing the determination of the activation energy [21,22,23,24].

**Figure 8:**
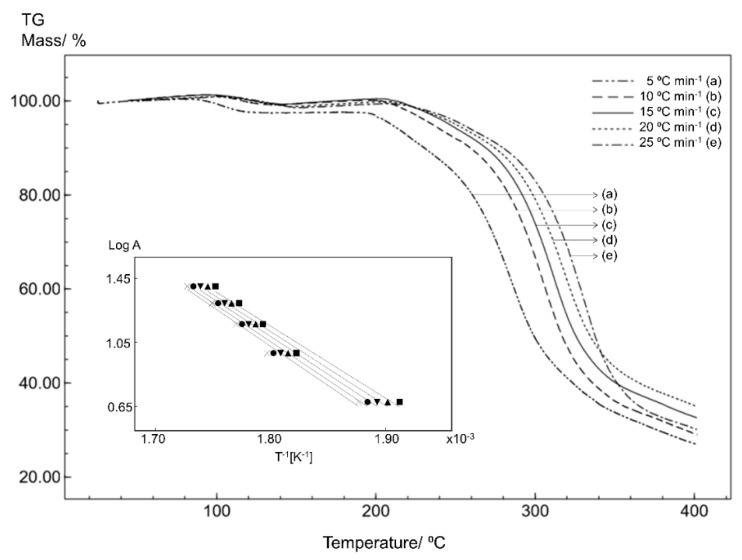
The TG curves for STG obtained for different heating rates with a synthetic air atmosphere (50 mL min^−1^). The inset shows the linear tendency of the correlation curves applying the Ozawa method.

**Figure 9:**
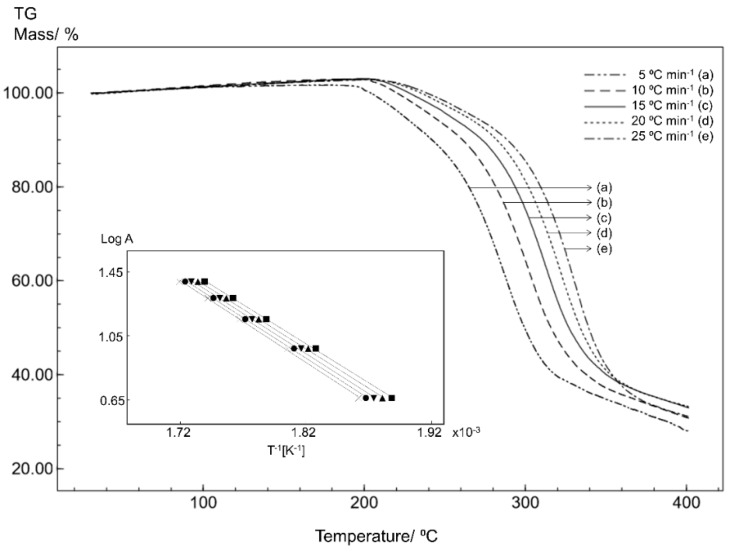
The TG curves for STGA obtained for different heating rates with a synthetic air atmosphere (50 mL min^−1^). The inset shows the linear tendency of the correlation curves applying the Ozawa method.

**Figure 10:**
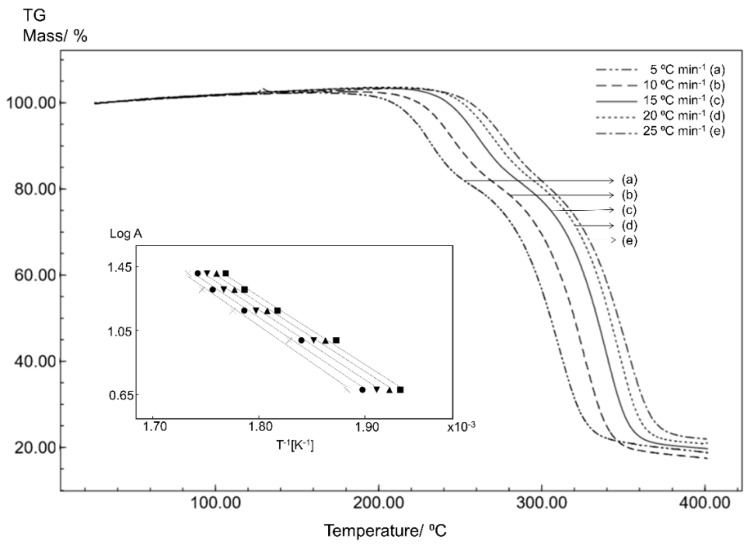
TG curves for STGB obtained for different heating rates with a synthetic air atmosphere (50 mL min^−1^). The inset shows the linear tendency of the correlation curves obtained applying the Ozawa method.

The kinetics results are shown in Figure 8, Figure 9 and Figure 10. Overlapping the thermogravimetric curves revealed a shift to higher temperatures as the heating rate increases and the inserts in the figures show correlation curves with a linear trend for all samples. 

The kinetics parameters obtained for STG (Figure 8), STGA (Figure 9) and STGB (Figure 10) are shown in Table 1.

The results for the kinetics parameters demonstrate a slightly higher activation energy for STG, suggesting that the dehydration process had little effect on the thermal stability of the drug. 

Furthermore, the kinetics study shows that STGB has a significantly lower activation energy than STG and STGA, indicating that the removal of the phosphate group from sitagliptin reduces the thermal stability of the sample.

### 3.4. Fourier Transform Infrared (FTIR) Spectroscopy

**Figure 11:**
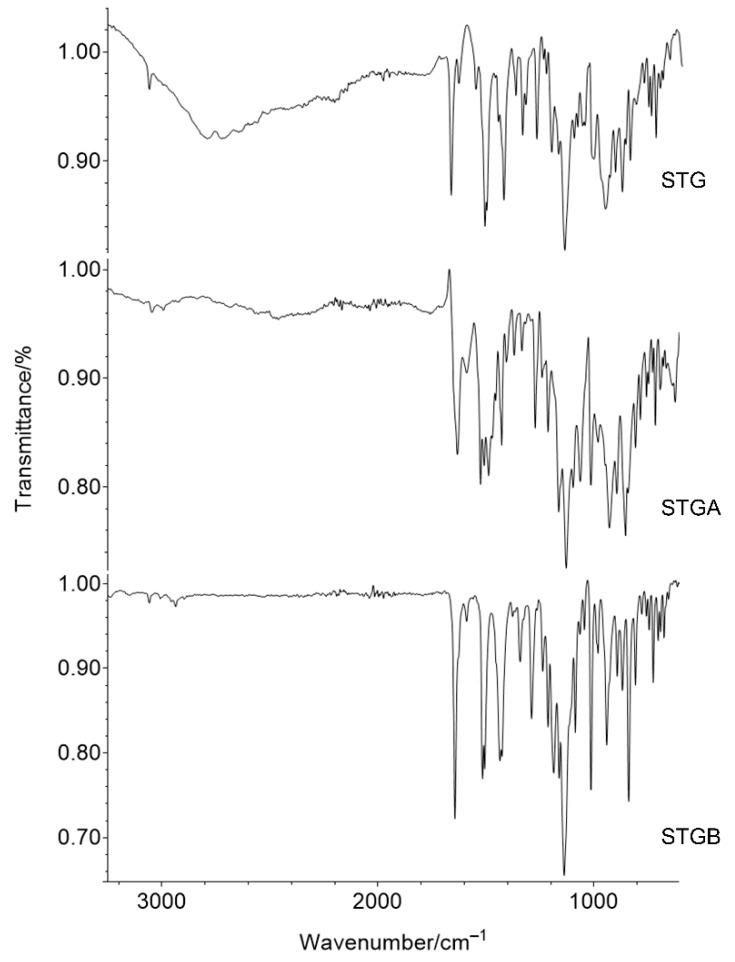
Comparison of Fourier transform infrared (FTIR) spectra for samples of sitagliptin phosphate monohydrate (STG), sitagliptin phosphate anhydrous (STGA) and sitagliptin base form (STGB).

The ATR-FTIR spectra obtained for the STG, STGA and STGB are shown in Figure 11. It can be observed that there are common vibrational bands for the three samples, which are associated with the major functional groups of sitagliptin. 

The band regions can be assigned as follows: 1465 cm^−1^ is related to alkane stretching (C–H), 1750–1735 cm^−1^ is associated with the C=O bond of carbonyl, and 1690–1640 cm^−1^ refers to the imine group (C=N). The vibration at 1250–1000 cm^−1^ is related to fluoride (C–F) and 3200–3100 cm^−1^ is associated with amine groups (NH_2_) [16,25,26,27,28].

The main difference between STG and the other two samples (STGA and STGB) was seen in the region between 3500 and 2500 cm^−1^, due to O–H stretching vibrations characteristic of water molecules, present only in the sitagliptin phosphate monohydrate molecule [16,29].

### 3.5. Raman Spectroscopy

Raman spectroscopy is a technique based on the principle of dispersion where the emission of monochromatic laser radiation causes vibrations in the sample molecule from the inelastic collision of the incident monochromatic radiation (laser) and the molecules present in the sample [30].

**Figure 12:**
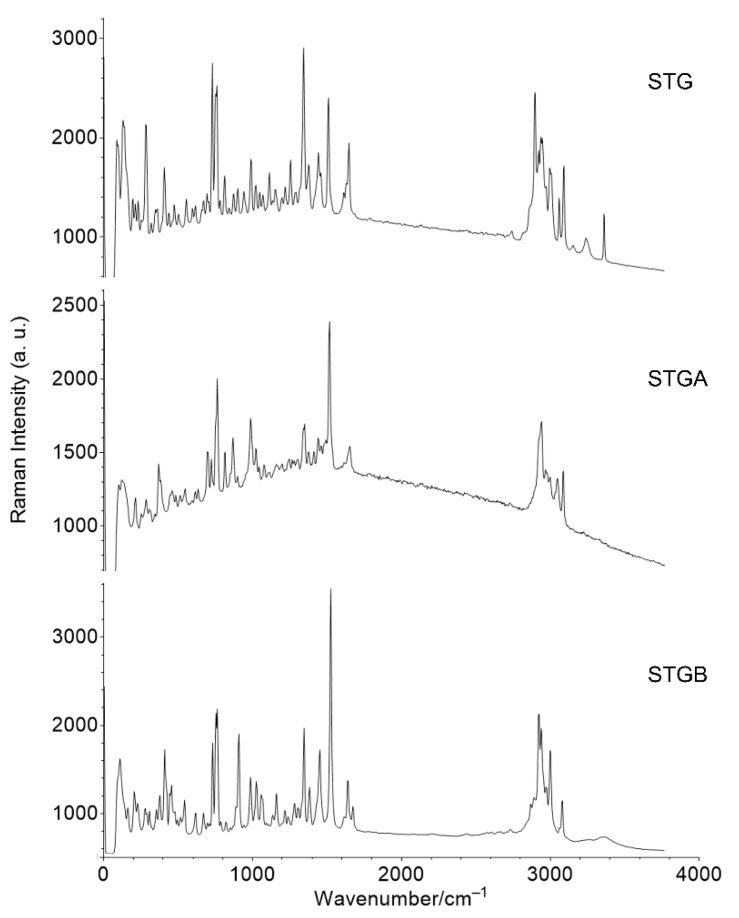
Raman spectra for sitagliptin phosphate monohydrate (STG), sitagliptin phosphate anhydrous (STGA) and sitagliptin base form (STGB).

Raman spectroscopy was performed on the STG, STGA, STGB samples and the results are shown in Figure 12. Based on the spectra, the characterization of the different forms of sitagliptin can be based on the stretching vibrations of C–H at 2950 cm^−1^, C–C at 1515–1525 cm^−1^, C=O at 1650 cm^−1^, C–N at 1345 cm^−1^ and N–H at 3077–3090 cm^−1^, along with the vibration related to the CF_3_ group at 1025–1060 cm^−1^ [31].

The major differences were observed in the O–H stretching vibrations at 3360 cm^−1^, present only in the STG spectrum (related to water molecules) and the presence of the phosphate group (P–O) stretching vibration at 815 cm^−1^, present only in the case of the STG and STGA forms. In fact, this result confirms the structure of the sitagliptin base form. 

### 3.6. X-ray Powder Diffraction (XRPD)

The diffraction of X-rays by a crystalline material following the direction of an incident X-ray beam toward a material with uniform atomic structure is caused by the constructive interference process. The XRPD technique was used to qualitatively identify the crystallinity of the samples [32].

**Figure 13:**
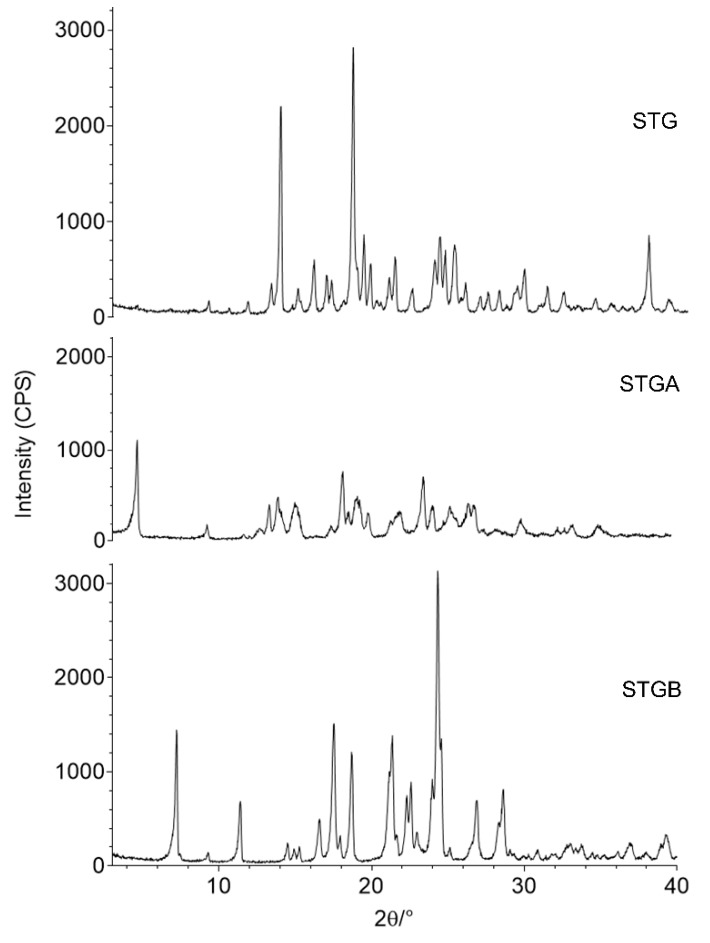
X-ray powder diffraction (XRPD) patterns for sitagliptin phosphate monohydrate (STG), sitagliptin phosphate anhydrous (STGA) and sitagliptin base form (STGB).

The XRPD results for STG, STGA and STGB are shown in Figure 13. According to the diffraction pattern, STG is a crystalline material, with major characteristic peaks at 13.2°, 13.8°, 15.9°, 18.4°, 19.1°, 21.2°, 24.0°, 25.0°, 25.7°, 29.5° and 30.9° ± 0.2°. 

The STGA sample also has crystalline character, the main characteristic peaks being at 4.6°, 9.3°, 13.4°, 13.9°, 15.0°, 18.2°, 19.2°, 19.9°, 21.4° 25.4° and 26.9° ± 0.2°. Dwivedi et al. (2015) observed similar diffraction patterns for STG and STGA.

The STGB showed several reflections corresponding to those described by Perlman (2009), as follows: 7.4°, 11.5°, 16.7°, 17.7°, 18.9°, 24.1°, 24.5°, 27.0°, 28.5° and 28.8° ± 0.2°.

Comparatively, the STGA patterns showed reduced crystallinity after the dehydration process, leading to a slightly more unstable molecule, as observed in the kinetics analysis. However, the anhydrous molecule (STGA) had greater solubility when compared to STG.

Based on the patterns obtained, the STGB has higher crystallinity, however, it has lower thermal stability, as observed in the thermal analysis, and lower solubility. Since a solvate is dissolved in water, free ions interact with the polar water molecules and these interactions provide pharmaceutical salts with greater solubility than the free form [33].

The solubility is directly associated with the drug bioavailability, the aqueous solubility of a drug is a key property for the drug absorption after oral administration and thus low solubility leads to limited oral bioavailability [8,9]. 

Considering that the synthetic route of STG generates solvates and hydrates [13], and the dehydration process is an expensive step in industrial scale. Thus, considering the slight difference between STG and STGA, the most suitable form for pharmaceutical purposes and the development of dosage forms is STG. 

### 3.7. Scanning Electron Microscopy (SEM)

Analysis by scanning electron microscopy (SEM) provides information on the sample morphology, including crystal size and shape, which has a direct influence on the physical characteristics and may influence in pharmaceutical operations and mechanical properties, such as processability (grinding, mixing, powder flow, compression, dissolution and lyophilization) [34].

**Figure 14:**
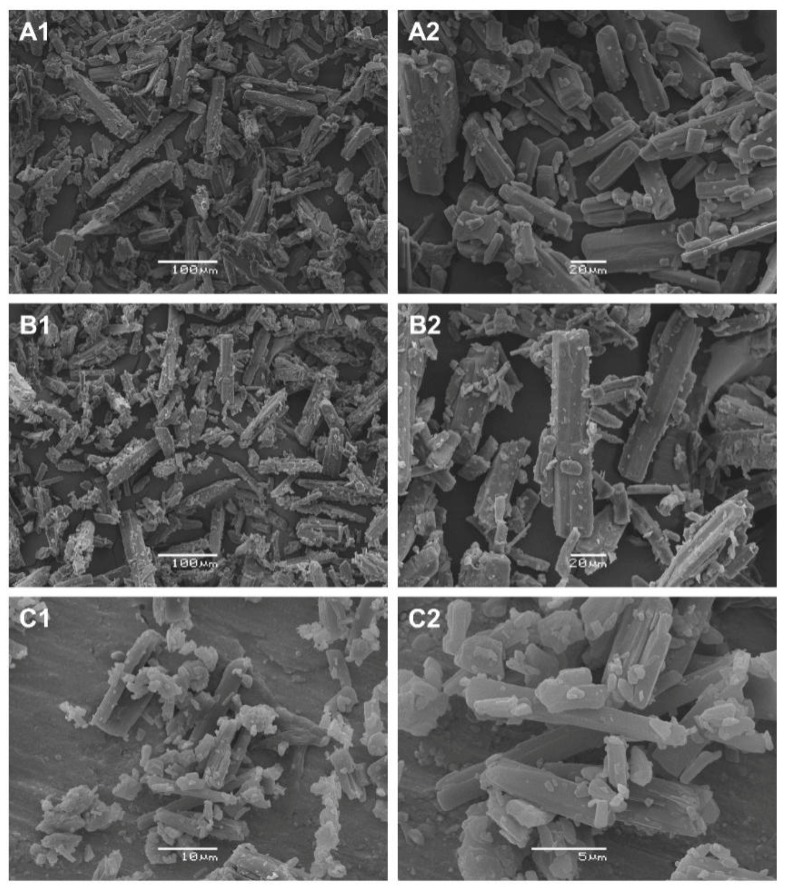
Photomicrographs obtained by scanning electron microscopy (SEM): STG (**A1** and **A2**; 200× and 600×), STGA (**B1** and **B2**; 200× and 600×) and STGB (**C1** and **C2**; 2000× and 5000×).

The micrographs for STG, STGA and STGB obtained by SEM are shown in Figure 14.

The sitagliptin phosphate monohydrate shown in Figure 14(A1,A2) can be classified as an orthorhombic crystal system. As described by Kaduk et al. (2015) [35], the structure of STG is comprised of flat and thin crystals (platy form). 

The microscopic evaluation of sitagliptin phosphate anhydrous (Figure 14(B1,B2)) revealed that the dehydration process did not alter the morphological characteristics compared with STG. The images C1 and C2 in Figure 14 show that the particles of the sitagliptin base form were smaller (by around a factor of 10) when compared to the other samples (note the magnification), but maintained the same shape and morphology.

## 4. Conclusions

The solid-state characterization of sitagliptin phosphate monohydrate (STG), sitagliptin phosphate anhydrous (STGA) and the sitagliptin base form (STGB) was carried out using several analytical techniques, such as DSC, TG, FTIR and RAMAN spectroscopy, XRPD and SEM. 

The thermoanalysis showed that after the dehydration of STG there was a crystalline transition, altering the melting point and solubility of the compound. The dehydration of STG was confirmed by TG–MS analysis, with the release of water at 117 °C. 

The crystalline behavior of the sitagliptin base form (STGB) differed from that of the STG and STGA, and the major difference was a lower fusion event. Specifically, the melting points were 120.29 °C for STGB, 206.37 °C for STG and 214.92 °C for STGA. 

During pre-formulation studies in the development of new dosage forms, it is essential to consider the influence of the different crystal structures of anhydrous and solvate compounds and the correlation with the solid-state properties. The results of this study highlight that STG is suggested to be the most suitable crystalline form for use in the development of safe and efficient new pharmaceutical dosage forms. 

## Figures and Tables

**Table 1:** Kinetics parameters obtained for STG, STGA and STGB in non-isothermal kinetics analysis.

	Activation Energy (E_a_)	Coefficient of Variation	Reaction Order
**STG**	89.29 ± 2.881 kJ mol^−1^	1.301%	n = 0
**STGA**	88.84 ± 1.561 kJ mol^−1^	1.757%	n = 0
**STGB**	78.21 ± 2.591 kJ mol^−1^	3.313%	n = 0
